# Supplementary material for: Extracellular Vesicle Molecular Signatures Characterize Metastatic Dynamicity in Ovarian Cancer
Source: Front Oncol. 2021 Nov 18;11:718408. doi: 10.3389/fonc.2021.718408 (PMC8637407; doi:10.3389/fonc.2021.718408)
Supplement: Supplementary file 1 [file DataSheet_1.pdf]

**Supplemental figure and table captions.**

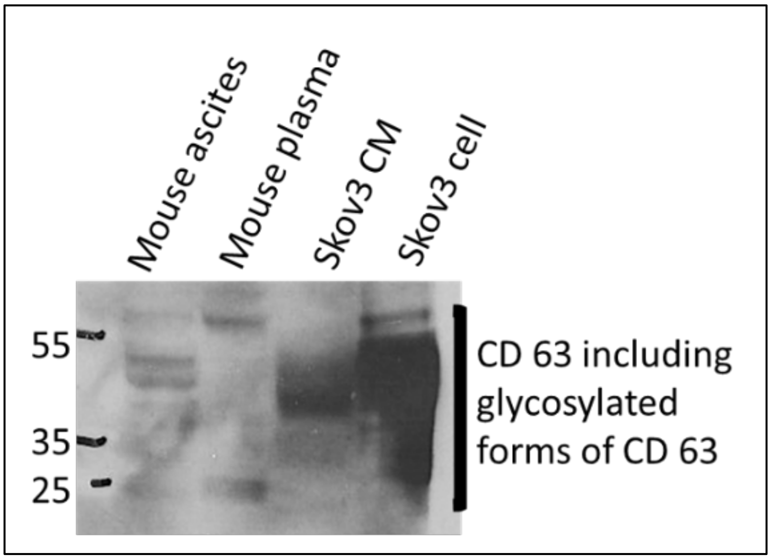


**Supplemental figure 1**: Expression of CD63 in exosomes isolated from mouse ascites (lane1), plasma (lane2) and SKOV3 cell culture medium (lane 3) and cell lysate (lane 4).

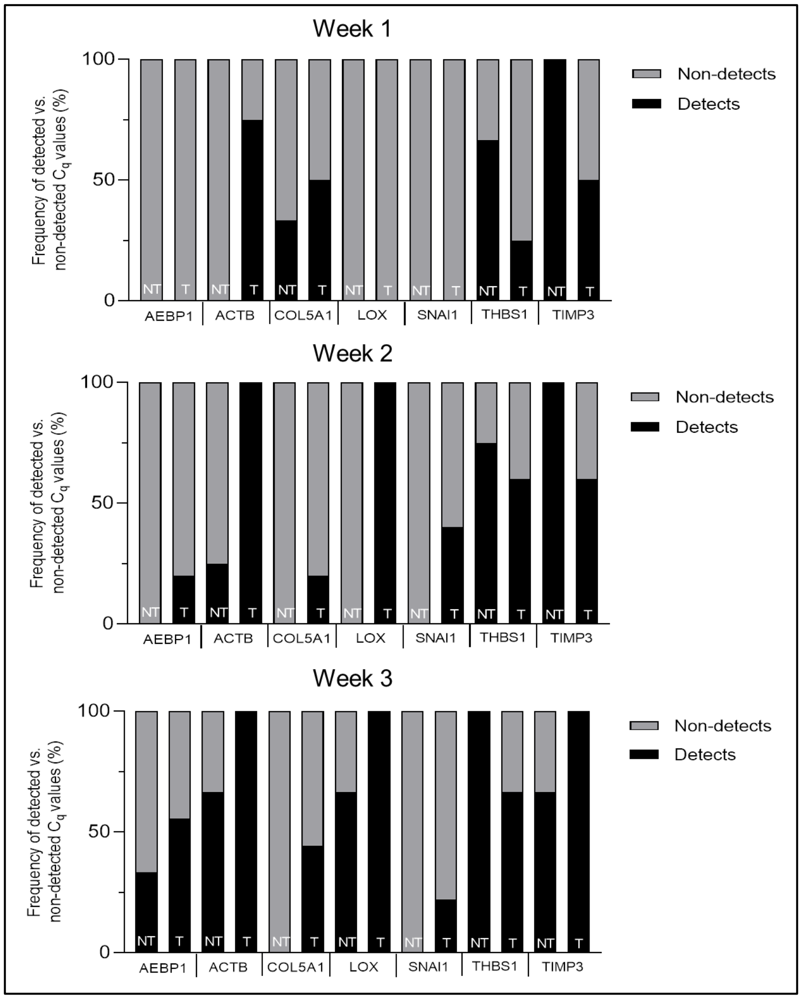


**Supplemental figure 2**: Frequency plots of detected vs. non-detected $C_q$ values in plasma-derived sEV samples.

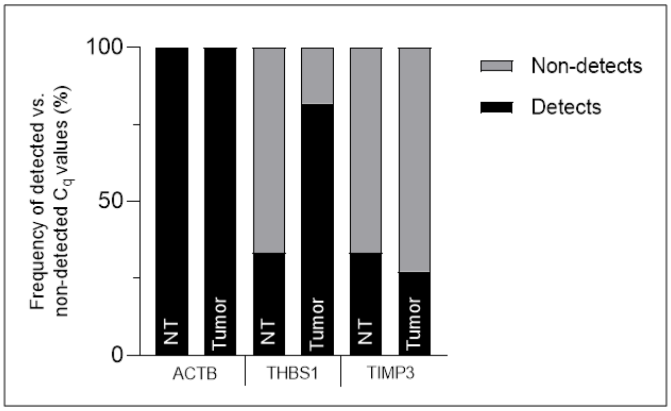


**Supplemental figure 3**: Frequency plots of detected vs. non-detected $C_q$ values in human plasma-derived sEV samples.

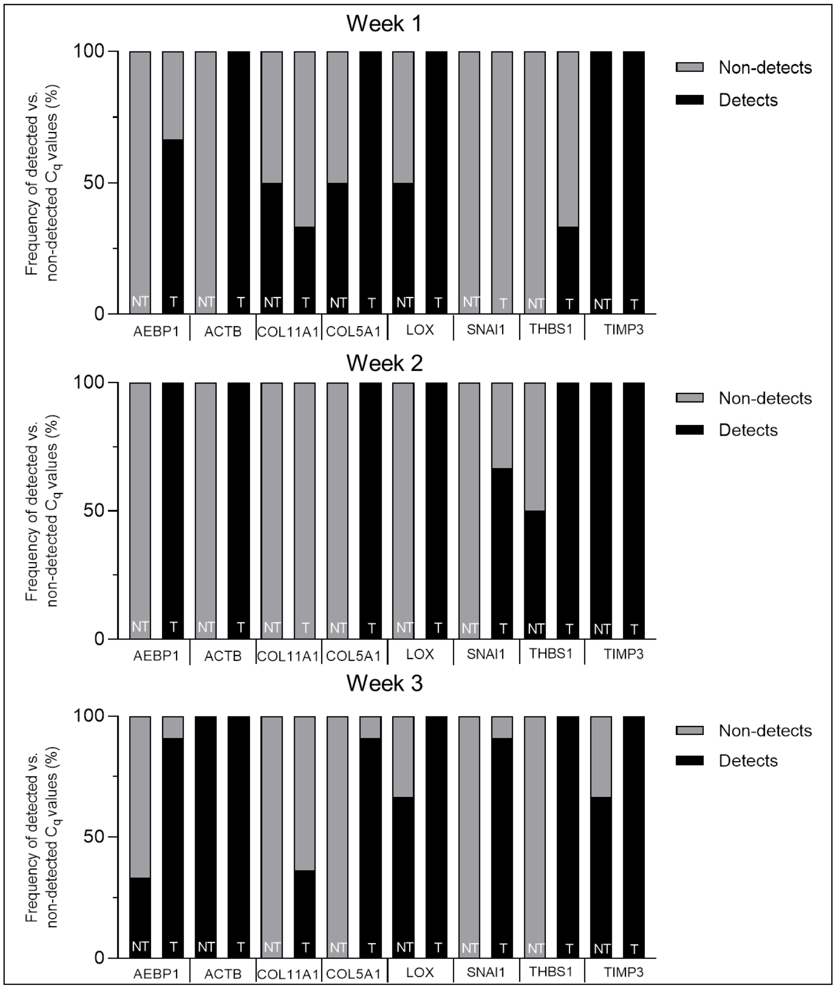


**Supplemental figure 4**: Frequency plots of detected vs. non-detected $C_q$ values in ascites-derived sEV samples.

**Supplemental table 1**: TaqMan gene expression assays used for qRT-PCR purchased from Thermo Fisher.

| **Gene Name** | **Gene expression assay ID** |
|---|---|
| *ACTB* | Hs99999903_m1 |
| *AEBP1* | Hs00937468_m1 |
| *COL5A1* | Hs00609088_m1 |
| *COL11A1* | Hs01097664_m1 |
| *GAPDH* | Hs02786624_g1 |
| *LOX* | Hs00942480_m1 |
| *NECTIN4* | Hs00363974_m1 |
| *POSTN* | Hs01566750_m1 |
| *SNAI1* | Hs00195591_m1 |
| *THBS1* | Hs00962908_m1 |
| *TIMP3* | Hs00165949_m1 |

**Supplemental table 2:** Datasets used for OvMARK genetic analysis.

| Gene Datasets | |
|---|---|
| GSE26712 | https://www.ncbi.nlm.nih.gov/geo/query/acc.cgi?acc=GSE26712 |
| GSE13876 | https://www.ncbi.nlm.nih.gov/geo/query/acc.cgi?acc=GSE13876 |
| GSE14764 | https://www.ncbi.nlm.nih.gov/geo/query/acc.cgi?acc=GSE14764 |
| GSE30161 | https://www.ncbi.nlm.nih.gov/geo/query/acc.cgi?acc=GSE30161 |
| GSE19161 | https://www.ncbi.nlm.nih.gov/geo/query/acc.cgi?acc=GSE19161 |
| GSE19829 | https://www.ncbi.nlm.nih.gov/geo/query/acc.cgi?acc=GSE19829 |
| GSE26193 | https://www.ncbi.nlm.nih.gov/geo/query/acc.cgi?acc=GSE26193 |
| GSE18520 | https://www.ncbi.nlm.nih.gov/geo/query/acc.cgi?acc=GSE18520 |
| GSE31245 | https://www.ncbi.nlm.nih.gov/geo/query/acc.cgi?acc=GSE31245 |
| GSE9899 | https://www.ncbi.nlm.nih.gov/geo/query/acc.cgi?acc=GSE9899 |
| GSE17260 | https://www.ncbi.nlm.nih.gov/geo/query/acc.cgi?acc=GSE17260 |
| GSE32062 | https://www.ncbi.nlm.nih.gov/geo/query/acc.cgi?acc=GSE32062 |
| TCGA | https://www.cancer.gov/about-nci/organization/ccg/research/structural-genomics/tcga |

**Supplemental table 3**: Differential expression of individual genes in the 10-gene panel correlates with disease-free survival. The OvMark algorithm was used to determine hazard ratios (>1 correlates with poor outcome, <1 correlates with good outcome-blue) and to show statistical significance between high and low expression.

| | **Expression** | |
|---|---|---|
| | **Hazard ratio** | **p value** |
| *ACTB* | 1.115 | 0.0356 |
| *AEBP1* | 1.238 | 4.09E-5 |
| *COL5A1* | 1.229 | 0.00502 |
| *COL11A1* | 1.276 | 9.19E-4 |
| *LOX* | 1.234 | 0.0042 |
| *NECTIN4* | 1.03 | 0.7211 |
| *POSTN* | 1.341 | 1.55E-08 |
| *SNAI1* | 0.9676 | 0.657 |
| *THBS1* | 1.278 | 8.39E-4 |
| *TIMP3* | 1.339 | 1.89E-15 |

**Supplemental table 4**: Differential expression of individual genes in the 10-gene panel correlates with disease-free survival in patients with serous ovarian cancer and endometrioid cancer. The OvMark algorithm was used to determine hazard ratios (>1 correlates with poor outcome, <1 correlates with good outcome-blue) and to show statistical significance between high and low expression.

| | **Serous** | | **Endometrioid** | |
|---|---|---|---|---|
| | **Hazard ratio** | **p value** | **Hazard ratio** | **p value** |
| *ACTB* | 1.202 | 0.0430 | 1.095 | 0.893 |
| *AEBP1* | 1.254 | 0.00447 | 1.327 | 0.673 |
| *COL5A1* | 1.306 | 0.00337 | 1.719 | 0.415 |
| *COL11A1* | 1.337 | 0.00146 | 1.181 | 0.814 |
| *LOX* | 1.279 | 0.000134 | 0.5114 | 0.173 |
| *NECTIN4* | 1.039 | 0.672 | 4.401 | 0.127 |
| *POSTN* | 1.394 | 0.00271 | 1.957 | 0.337 |
| *SNAI1* | 1.007 | 0.894 | 1.148 | 0.837 |
| *THBS1* | 1.332 | 0.001676 | 0.774 | 0.7172 |
| *TIMP3* | 1.399 | 0.000228 | 1.24 | 0.761 |

**Supplemental table 5**: Differential expression of individual genes in the 10-gene panel correlates with disease-free survival in various stages of ovarian cancer development. The OvMark algorithm was used to determine hazard ratios (>1 correlates with poor outcome, <1 correlates with good outcome-blue) and to show statistical significance (red) between high and low expression.

| | **grade1** | | **grade2** | | **grade3** | |
|---|---|---|---|---|---|---|
| | **Hazard ratio** | **p value** | **Hazard ratio** | **p value** | **Hazard ratio** | **p value** |
| *ACTB* | 1.311 | 0.326 | 1.094 | 0.516 | 1.029 | 0.726 |
| *AEBP1* | 1.617 | 0.222 | 1.2 | 0.185 | 1.239 | 0.00891 |
| *COL5A1* | 1.365 | 0.421 | 1.151 | 0.307 | 1.280 | 0.0338 |
| *COL11A1* | 1.815 | 0.116 | 1.163 | 0.273 | 1.388 | 0.0474 |
| *LOX* | 1.856 | 0.104 | 1.081 | 0.571 | 1.242 | 0.0626 |
| *NECTIN4* | 1.089 | 0.824 | 0.9758 | 0.860 | 0.9641 | 0.753 |
| *POSTN* | 2.801 | 0.00464 | 1.267 | 0.0843 | 1.433 | 0.00203 |
| *SNAI1* | 1.379 | 0.402 | 1.102 | 0.482 | 0.9713 | 0.802 |
| *THBS1* | 1.782 | 0.1623 | 1.205 | 0.1747 | 1.272 | 0.03815 |
| *TIMP3* | 2.385 | 0.0181 | 1.269 | 0.0823 | 1.279 | 0.0339 |

**Supplemental table 6:** Patient characteristics.

| | Non-metastatic (n=6) | Metastatic (n=5) | Overall (n=11) |
|---|---|---|---|
| **Age, years (median, range)** | 56 (50-77) | 56.5 (38-79) | 56 (38-79) |
| **Histology** | | | |
| **Papillary serous** | 1 | 3 | 4 |
| **Clear cell** | 2 | 1 | 3 |
| **Endometrioid** | 2 | 0 | 1 |
| **Mixed** | 1 | 1 | 2 |
| **Differentiation** | | | |
| **Well or moderately** | 5 | 1 | 6 |
| **Poorly** | 1 | 1 | 2 |
| **Unknown** | 1 | 3 | 4 |
| **Organ involvement** | | | |
| **Ovary (bilateral)** | 1 | 4 | 5 |
| **Ovary (unilateral)** | 5 | 1 | 6 |
| **Tubal involvement** | 1 | 4 | 5 |
| **Peritoneum** | 1 | 5 | 6 |
| **Uterus** | 1 | 2 | 3 |
| **Additional organs** | 0 | 1 | 1 |
